# [^68^ Ga]Ga-FAPI-04 PET/CT may be a predictor for early treatment response in rheumatoid arthritis

**DOI:** 10.1186/s13550-023-01064-4

**Published:** 2024-01-04

**Authors:** Qingqing Pan, Huaxia Yang, Ziyue Zhou, Min Li, Xu Jiang, Fang Li, Yaping Luo, Mengtao Li

**Affiliations:** 1 Department of Nuclear Medicine, Chinese Academy of Medical Sciences and Peking Union Medical College Hospital, No.1 Shuaifuyuan Wangfujing, Dongcheng District, Beijing, 100730 China; 2 Beijing Key Laboratory of Molecular Targeted Diagnosis and Therapy in Nuclear Medicine, Beijing, 100730 China; 3 Department of Rheumatology and Clinical Immunology, National Clinical Research Center for Dermatologic and Immunologic Diseases, the Ministry of Education Key Laboratory, Chinese Academy of Medical Sciences and Peking Union Medical College Hospital, Beijing, China; 4 State Key Laboratory of Difficult, Severe and Rare Diseases, Chinese Academy of Medical Sciences and Peking Union Medical College Hospital, Beijing, China; 5 Department of Endocrinology and Rheumatology, Taihe Hospital, Hubei University of Medicine, Shiyan, Hubei China; 6 State Key Laboratory of Common Mechanism Research for Major Diseases, Beijing, China; 7 Medical Research Center, Peking Union Medical College Hospital, Chinese Academy of Medical Sciences, Beijing, China

**Keywords:** Rheumatoid arthritis, [^68^ Ga]Ga-FAPI-04, [^18^F]FDG, PET/CT, Treatment response

## Abstract

**Background:**

The identification of biomarkers predicting the treatment response of rheumatoid arthritis (RA) is important. [^68^ Ga]Ga-FAPI-04 showed markedly increased uptake in the joints of patients with RA. The purpose of this study is to investigate whether [^68^ Ga]Ga-FAPI-04 PET/CT can be a predictor of treatment response in RA.

**Results:**

Nineteen patients diagnosed with RA in the prospective cohort study were finally enrolled. Both total synovitis uptake (TSU) and metabolic synovitis volume (MSV) in [^68^ Ga]Ga-FAPI-04 and [^18^F]FDG PET/CT of the responders were significantly higher than those in non-responders according to Clinical Disease Activity Index (CDAI) and Simplified Disease Activity Index (SDAI) response criteria at 3-months’ follow-up (*P* < 0.05). The PET joint count (PJC) detected in [^68^ Ga]Ga-FAPI-04 and [^18^F]FDG PET/CT were also significantly higher in CDAI responders than non-responders (*P* = 0.016 and 0.045, respectively). The clinical characteristics of disease activity at baseline did not show significant difference between the responders and non-responders, except CRP (*P* = 0.035 and 0.033 in CDAI and SDAI response criteria, respectively). The baseline PJC_FAPI_, TSU_FAPI_ and MSV_FAPI_ > cutoff values in [^68^ Ga]Ga-FAPI-04 PET/CT successfully discriminated CDAI and SDAI responders and non-responders at 3-months’ follow-up.

**Conclusion:**

[^68^ Ga]Ga-FAPI-04 uptake at baseline were significantly higher in early responders than those in non-responders.

*Trial registration* ClinicalTrials. NCT04514614. Registered 13 August 2020, https://register.clinicaltrials.gov/prs/app/action/SelectProtocol?sid=S000A4PN&selectaction=Edit&uid=U0001JRW&ts=2&cx=-x9t7cp

## Background

Rheumatoid arthritis (RA) is a chronic systemic autoimmune inflammatory disorder that primarily involves synovial joints. Early use of disease-modifying antirheumatic drugs (DMARDs) and efforts toward tight control with a treat-to-target strategy are essential to the good control of synovitis and prevention of joint injury [1]. The expanding array of drugs available for treating RA is creating challenges in drug selection for the individual patient. Some of the patients do not have good response to these drugs, for example, the remission rate of methotrexate ranges between 30 and 50% [2], while 30 to 40% of patients have unsatisfactory control of disease symptoms with biological DMARDs [3]. The efficacy also varies over time in individual patients [4]. Apart from this, the potential side effects of these agents, such as hepatotoxicity from methotrexate [5], infusion reactions and risk of infection [6], as well as their cost and accessibility should also be considered when making treatment plan [7]. Thus the identification of biomarkers that would predict the treatment response prior to drug exposure is therefore a current priority. Antibodies to citrullinated peptides, rheumatoid factor, and the interferon signature are the most robust validated biomarkers identified to date [4]. However, the complexity of the treatment response in a given patient and substantial variability across patients suggest that biomarkers may be more helpful in combination than singly.

The imaging techniques for assessing RA, including musculoskeletal ultrasound, MRI and positron emission tomography/computed tomography (PET/CT), may be more sensitive and specific than clinical exam to reveal subclinical inflammation. As to disease pathogenesis of RA, an increasing role is attributed to fibroblast-like synoviocyte, which contribute to the pannus formation and destruction of articular cartilage and bones [8]. Recently, fibroblast-like synoviocyte targeted treatment showed promising results for RA therapy in preclinical animal models and early phase trials of RA patients [9–13]. Fibroblast activation protein (FAP), a type II cell surface serine protease, was demonstrated to be overexpressed in activated synovial fibroblasts [14, 15]. Preclinical studies of immuno-PET and immuno-SPECT with radiolabeled anti-FAP antibody also showed high tracer accumulation in the arthritic joints in murine experimental RA [16–18]. Recently we have conducted a prospective cohort study to verify the use of FAP-targeted PET/CT imaging in patients with RA. We imaged the arthritic joints in RA patients with [^68^ Ga]Ga-fibroblast activation protein inhibitor-04 ([^68^ Ga]Ga-FAPI-04), a recently introduced FAP-targeted PET agent, and found markedly increased uptake of [^68^ Ga]Ga-FAPI-04 in the arthritic joints of patients with RA. The positive joint count detected in [^68^ Ga]Ga-FAPI-04 PET/CT was found to be positively correlated with clinical disease activity variables [19]. Further in this study, we wonder whether [^68^ Ga]Ga-FAPI-04 PET/CT can be used as a predictor of treatment response in RA.

## Methods

### Study design and patients

**Fig. 1:**
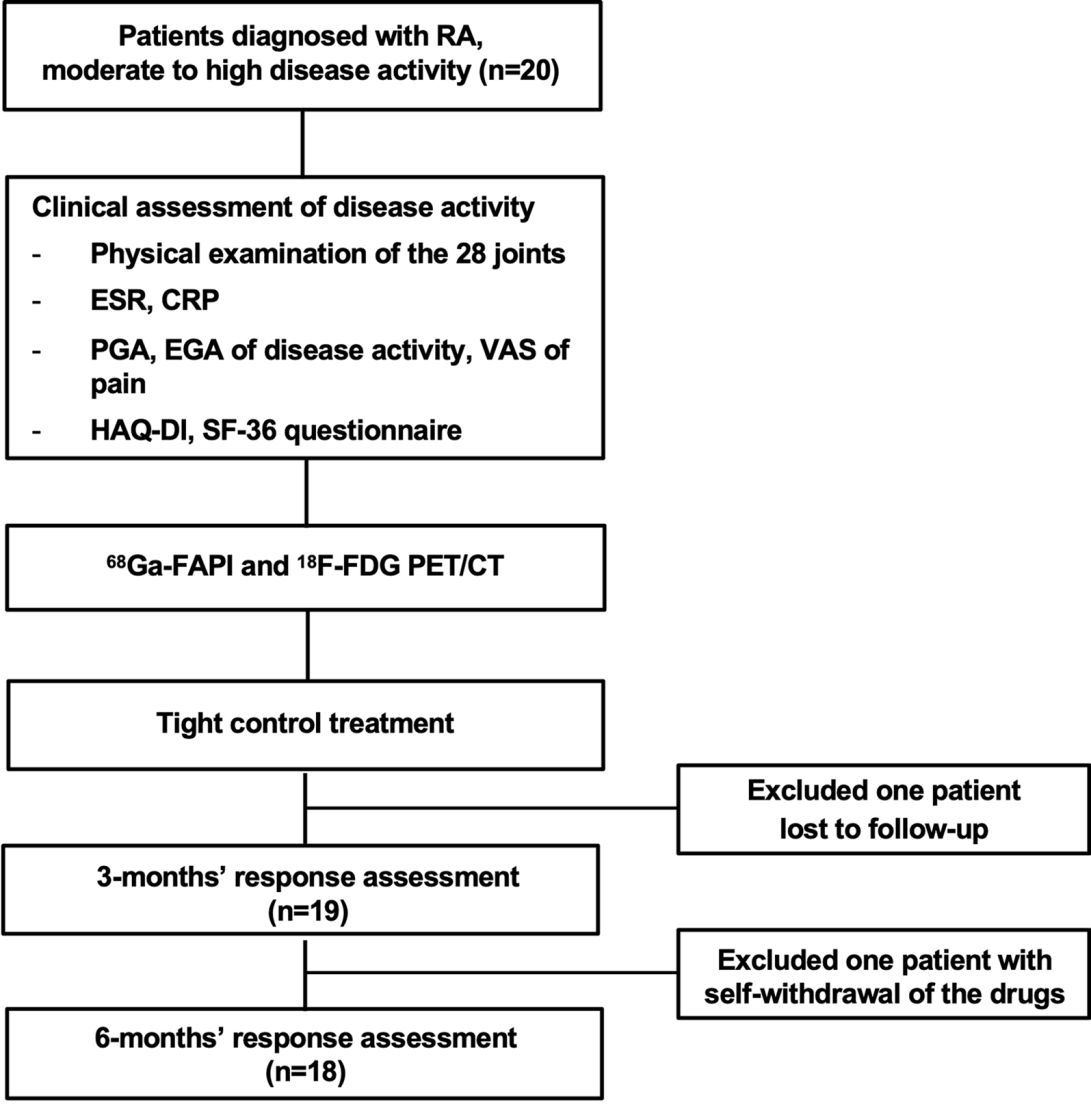
Flowchart of enrollment and study profile. *RA* Rheumatoid arthritis, *ESR* Erythrocyte sedimentation rate, *CRP* C-reactive protein, *VAS* Visual analog scale, *PGA* Patient global assessment, *EGA* Evaluator global assessment, *HAQ-DI* Health Assessment Questionnaire Disability Index, *SF-36* Short Form-36.

This is a retrospective analysis of the data from our prospective cohort study on [^68^ Ga]Ga-FAPI-04 PET/CT in evaluation of RA (NCT 04514614). The study was approved by the Institutional Review Board of Peking Union Medical College Hospital (protocol ZS-1810). Written informed consent was obtained from each patient. Twenty patients diagnosed with RA with moderate to high disease activity in the Department of Rheumatology and Clinical Immunology of Peking Union Medical College Hospital were consecutively recruited from Sep 2020 to Dec 2021. At enrollment, the disease activity of RA was assessed clinically by two experienced rheumatologists (HY and ML). Then the patients were referred for [^68^ Ga]Ga-FAPI-04 PET/CT for evaluation of the disease, and 2- [^18^F]fluoro-2-deoxy-d-glucose ([^18^F]FDG) PET/CT was done as a reference. Then the patients underwent tight control treatment against RA. Nineteen patients among them were followed up for treatment response at 3 months’ and 6 months’ time points after initiation of treatment, and they were finally enrolled in the current study. One patient that was lost to follow up of response was excluded from the study. The flowchart of patients’ enrollment and study profile was shown in Fig. 1.

### Clinical assessment of disease activity and response criteria

Clinical assessment of disease activity was performed at enrollment, 3-months and 6-months’ follow-up after initiation of treatment. The core variables of disease activity include: (1) physical examination of the joints, including tender joint count (TJC, pain on pressure or motion) and swollen joint count (SJC, soft tissue swelling and effusion), using 28-joint counts (shoulder, elbow, wrist, metacarpophalangeal joint, proximal interphalangeal joint, knee) [20]; (2) erythrocyte sedimentation rate (ESR) and C-reactive protein (CRP) as measures of acute-phase response; (3) patient assessment of pain, patient global assessment (PGA) and evaluator global assessment (EGA) of disease activity with visual analog scales (VAS); 4) patient self-report questionnaire of physical function assessment, including Health Assessment Questionnaire Disability Index (HAQ-DI) and the Medical Outcomes Study Short Form-36 (SF-36). The composite indices for disease activity assessment were calculated, including Clinical Disease Activity Index (CDAI, a numerical sum of TJC, SJC, PGA and EGA), Simplified Disease Activity Index (SDAI, the sum of CDAI and CRP level), and Disease Activity Score using 28-joint counts (DAS28) either with ESR or CRP [20]. Treatment response was evaluated according to CDAI and SDAI response criteria at 3-months and 6-months’ time points after initiation of treatment. The response criteria is defined according to the decrease of CDAI or SDAI compared to the baseline scores: major response, decrease ≥ 85%; moderate response, decrease ≥ 70%; minor response, decrease ≥ 50%; no response, decrease < 50% [21].

### PET/CT imaging

The DOTA-FAPI-04 peptide was purchased from CSBio Co (Menlo Park, CA). The radiolabeling of [^68^ Ga]Ga-FAPI-04 was performed manually before injection according to the procedures as previously published [22]. [^18^F]FDG was synthesized in-house with an 11 MeV cyclotron (CTI RDS 111, Siemens, Germany). The PET scans were performed on dedicated PET/CT scanners (Biograph 64 Truepoint TrueV, Siemens, Germany; Polestar m660, SinoUnion, China). For [^18^F]FDG PET/CT, the patients fasted for over 6 h, and the blood glucose levels were monitored (4.5–7.8 mmol/L) prior to an injection of [^18^F]FDG (5.55 MBq/kg). The PET/CT images (2 min/bed) were acquired with an uptake time of 75.6 ± 15.1 min. For [^68^ Ga]Ga-FAPI-04 PET/CT, imaging was performed (2 min/bed) with an uptake time of 51.2 ± 13.2 min after an injection of 103.6 ± 33.3 MBq [^68^ Ga]Ga-FAPI-04. The PET/CT scan was obtained from the tip of the skull to the crus. The acquired data were reconstructed using the ordered-subset expectation maximization method.

### Image interpretation

Two experienced nuclear medicine physicians (YL and QP) reviewed the PET/CT images, and were in consensus for the image interpretation. Increased articular radioactivity compared with the background uptake was defined as a positive joint. The number of PET positive joints was recorded as PET joint count (PJC). Quantitative measurement of 28-joint synovitis burden was measured as metabolic synovitis volume (MSV_FDG_ for [^18^F]FDG and MSV_FAPI_ for [^68^ Ga]Ga-FAPI-04, defined as the sum of the metabolic volumes of all 28-joint) and total synovitis uptake (TSU_FDG_ for [^18^F]FDG and TSU_FAPI_ for [^68^ Ga]Ga-FAPI-04, defined as the sum of individual MSV multiplied by its mean SUV). PET/CT data were transferred in DICOM format to MIM workstation (version 6.6.11, MIM Software, USA). Then volumes of interest were drawn including all 28-joint areas in the PET/CT images. Subsequently, joints contours were first semiautomatically segmented with a SUV cutoff of 2.5, and were then checked and manually adjusted to exclude the physiological uptakes such as injection sites, etc. Afterward, volumetric parameters of TSU_FAPI_, MSV_FAPI_, TSU_FDG_, MSV_FDG_ and SUVmax were automatically obtained from the statistics generated with the final volumetric extraction.

### Statistical analysis

Statistical analyses were done with Medcalc (version 19.6.4) and SPSS Statistics software (version 22.0, IBM SPSS Inc.). For correlation analyses, Pearson correlation coefficients (for data with normal distribution) or Spearman’s rank correlation coefficients (for skewed data) were conducted. Baseline characteristics between responders and non-responders were compared using t test for data with normal distribution and Wilcoxon test for skewed data (Shapiro–Wilk test for normality). Receiver-operating-characteristic (ROC) curve analyses were performed to predict responders. A* p*-value < 0.05 was considered statistically significant.

## Results

### Baseline clinical characteristics and PET/CT

Nineteen patients with RA (5 men and 14 women; 55.7 ± 9.8 yr, range 25–73 yr) were included in this study. Nine patients had new-onset RA that was treatment-naïve; the rest 10 patients had a history of RA and had recurrent or active disease at enrollment. The median disease duration was 48.0 months (range 1–372 months). All the patients were grading as being with high or moderate disease activity according to CDAI, SDAI, and DAS-28.

Both [^68^ Ga]Ga-FAPI-04 and [^18^F]FDG PET/CT were visually positive for detecting arthritis in all recruited patients. Among the 28-joint areas (532 joints in total), [^68^ Ga]Ga-FAPI-04 PET/CT detected 216 positive joints, whereas 201/216 joints were [^18^F]FDG avid. The rest 316 joints were negative in both [^68^ Ga]Ga-FAPI-04 and [^18^F]FDG PET/CT. The SUVmax of the most affected joint in each patient was higher in [^68^ Ga]Ga-FAPI-04 than in [^18^F]FDG PET/CT (SUVmax, 9.1 ± 4.6 vs 5.9 ± 2.9, respectively; *P* < 0.001). The TSU_FAPI_ and MSV_FAPI_ of [^68^ Ga]Ga-FAPI-04 PET/CT were 524.8 ± 552.4 SUV bw*ml and 130.9 ± 134.7 ml, respectively. In [^18^F]FDG PET/CT, the TSU_FDG_ and MSV_FDG_ were 271.1 ± 332.8 SUV bw*ml and 77.9 ± 86.1 ml, respectively. Both TSU and MSV in [^68^ Ga]Ga-FAPI-04 and [^18^F]FDG PET/CT were positively correlated with CRP level (TSU_FAPI_, r = 0.70, *P* = 0.001; MSV_FAPI_, r = 0.62, *P* = 0.005; TSU_FDG_, r = 0.70, *P* = 0.001; MSV_FDG_, r = 0.72, *P* = 0.001). Patients’ clinical characteristics and PET/CT measurements are shown in Table 1.

**Table 1:** Clinical characteristics and PET/CT measurements in RA patients

Characteristics	Value
Age (years; mean ± SD)	55.7 ± 9.8
Gender	5 males, 14 females
Erythrocyte sedimentation rate (ESR, mm/h)	58.0 ± 29.1
C-reactive protein (CRP, mg/dL)	3.8 ± 4.3
Tender joint counts (TJC)*	10.5 ± 4.9
Swollen joint counts (SJC)*	8.8 ± 4.7
VAS of pain	6.3 ± 1.4
PGA of disease activity	6.0 ± 1.3
EGA of disease activity	7.0 ± 1.1
HAQ-DI	1.7 ± 0.8
SF-36	46.6 ± 9.3
CDAI*	32.4 ± 9.8
SDAI*	36.1 ± 12.6
DAS28-ESR*	6.1 ± 0.9
DAS28-CRP*	5.6 ± 1.3
TSU_FAPI_ (SUV bw*ml)	524.8 ± 552.4
MSV_FAPI_ (mL)	130.9 ± 134.7
SUV_max_ of ^68^ Ga-FAPI PET/CT	9.1 ± 4.6
TSUG_FDG_ (SUV bw*ml)	271.1 ± 332.8
MSV_FDG_ (mL)	77.9 ± 86.1
SUVmax of ^18^F-FDG PET/CT	5.9 ± 2.9

^*^Assessed using 28-joint counts

*VAS* Visual analog scale, *PGA* Patient global assessment, *EGA* Evaluator global assessment, *HAQ-DI* Health Assessment Questionnaire Disability Index, *SF-36* Short Form-36, *CDAI* Clinical Disease Activity Index, *SDAI* Simplified Disease Activity Index, *DAS28* Disease Activity Score with 28-joint counts, *TSU* Total synovitis uptake, *MSV* Metabolic synovitis volume

### Response of tight control treatment

After baseline clinical assessment and PET/CT, treat-to-target with DMARDs was initiated. Nineteen patients received conventional synthetic DMARDs (including methotrexate, leflunomide, cyclophosphamide, and tripterygium wilfordii), and 12 patients had regimens containing biological or targeted synthetic DMARDs (including etanercept, adalimumab and Janus Kinase inhibitors). Six patients received additional prednisone, and six patients had non-steroidal anti-inflammatory drugs. At 3-months’ follow-up, DAS28-ESR, DAS28-CRP, CDAI and SDAI declined with a median percentage of 31.0%, 32.0%, 50.0% and 53.2%, respectively. According to CDAI response, 10 patients achieved response (major response in 3 patients, moderate response in 2 patients, minor response in 5 patients), and 9 patients were non-responders. According to SDAI response, 11 patients were responders (major response in 2 patients, moderate response in 4 patients, minor response in 5 patients), and 8 patients were non-responders.

After 3-months’ follow-up, one patient who achieved major response had self-withdrawal of the drugs, so her disease activity exacerbated and was excluded from the analysis of 6-months’ follow-up. At 6-months’ time point, the median decrease of DAS28-ESR, DAS28-CRP, CDAI and SDAI from baseline were 46.0%, 42.0%, 69.1% and 71.1%, respectively. According to CDAI response, 6 of the 9 non-responders at 3-months’ follow-up achieved response at 6 months; while according to SDAI response, 4 of the 8 non-responders at 3-months had treatment response at 6-months’ follow-up. Among the responders at 3-months, one patient experienced deterioration of disease activity and became non-responder at 6-months’ follow-up. The change of patients’ disease activity during treatment was listed in Table 2.

**Table 2:** The change of RA patients’ disease activity during treatment

	Enrollment	3-months	6-months
Interval between initiation of treatment and follow-up (months, IQR)	NA	2.8 (2.6, 3.6)	6.0 (5.7, 7.5)
CDAI	32.4 ± 9.8	16.2 ± 11.9	10.7 ± 9.3
SDAI	36.1 ± 12.6	18.2 ± 13.3	11.9 ± 9.7
DAS28-ESR	6.1 ± 0.9	4.3 ± 1.4	3.6 ± 1.3
DAS28-CRP	5.6 ± 1.3	3.9 ± 1.4	3.2 ± 1.2

*CDAI* Clinical Disease Activity Index, *SDAI* Simplified Disease Activity Index, *DAS28* Disease Activity Score with 28-joint counts

### PET/CT features in predicting responders

**Fig. 2:**
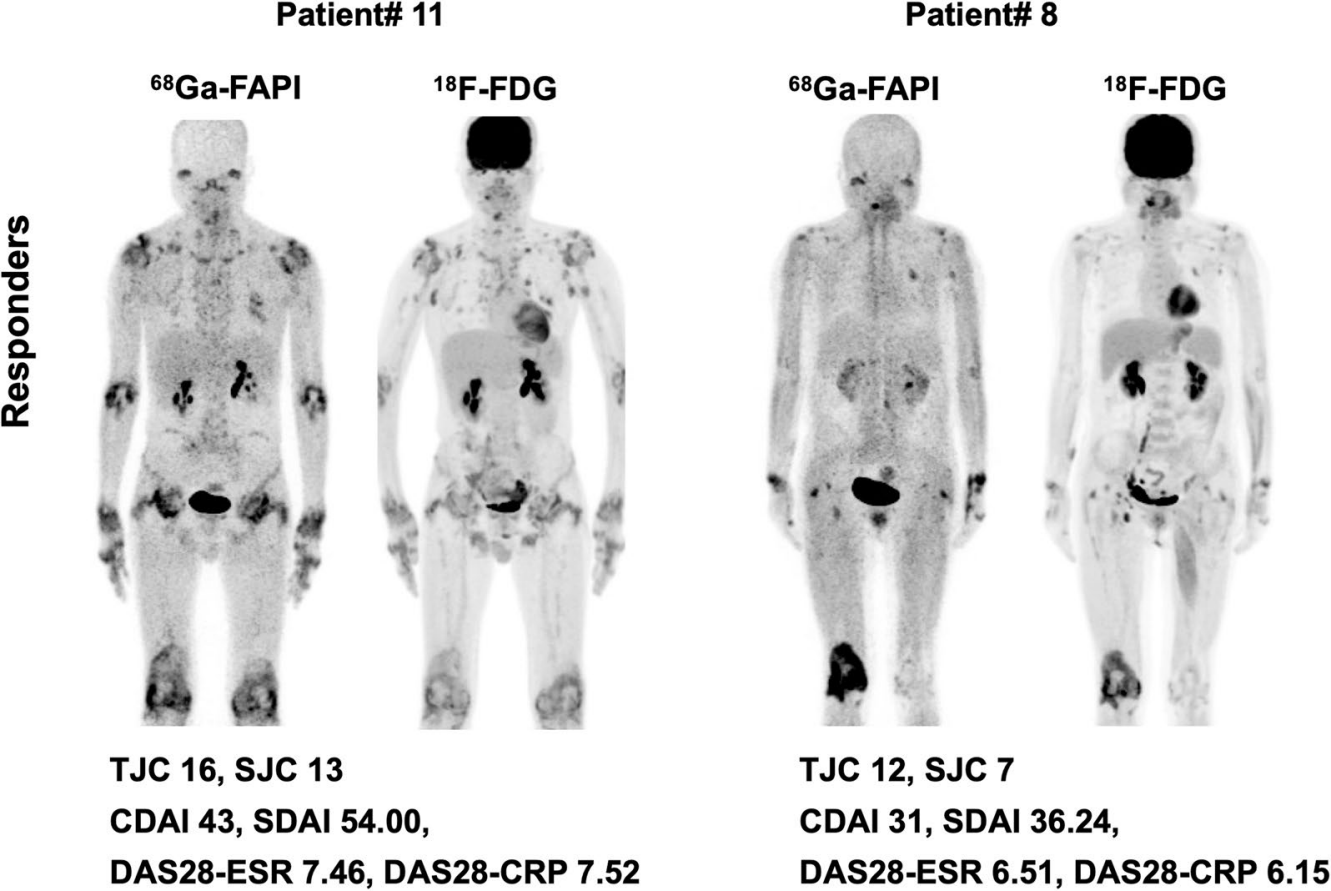
Representative [^68^ Ga]Ga-FAPI-04 and [^18^F]FDG PET/CT images of responders at 3-months’ follow-up based on CDAI and SDAI response criteria. Patient# 11 was a 50-year-old man with 28 affected joints detected in PET/CT (PJC_FAPI_ = 28, TSU_FAPI_ = 1620.0 SUV bw*ml, MSV_FAPI_ = 490.8 mL, TSU_FDG_ = 668.0 SUV bw*ml, MSV_FDG_ = 225.1 mL). He was treated with Tripterygium wilfordii, methotrexate, adalimumab, and prednisone. The CDAI and SDAI decreased 67.4% and 71.3% respectively from baseline at 3-months’ follow up. In patient# 8 (65-year-old woman), [^68^ Ga]Ga-FAPI-04 PET/CT showed 12 affected joints (PJC_FAPI_ = 12, TSU_FAPI_ = 1527.2 SUV bw*ml, MSV_FAPI_ = 294.1 mL, TSU_FDG_ = 696.73 SUV bw*ml, MSV_FDG_ = 166.6 mL). The CDAI and SDAI decreased 64.5% and 69.0% from baseline after 3 months treating with Tripterygium wilfordii and methotrexate. *ESR* Erythrocyte sedimentation rate, *CRP* C-reactive protein, *TJC* Tender joint count, *SJC* Swollen joint count, *CDAI* Clinical Disease Activity Index, *SDAI* Simplified Disease Activity Index, *DAS28* Disease Activity Score with 28-joint counts, *PJC* PET joint count, *TSU* Total synovitis uptake, *MSV* Metabolic synovitis volume

**Fig. 3:**
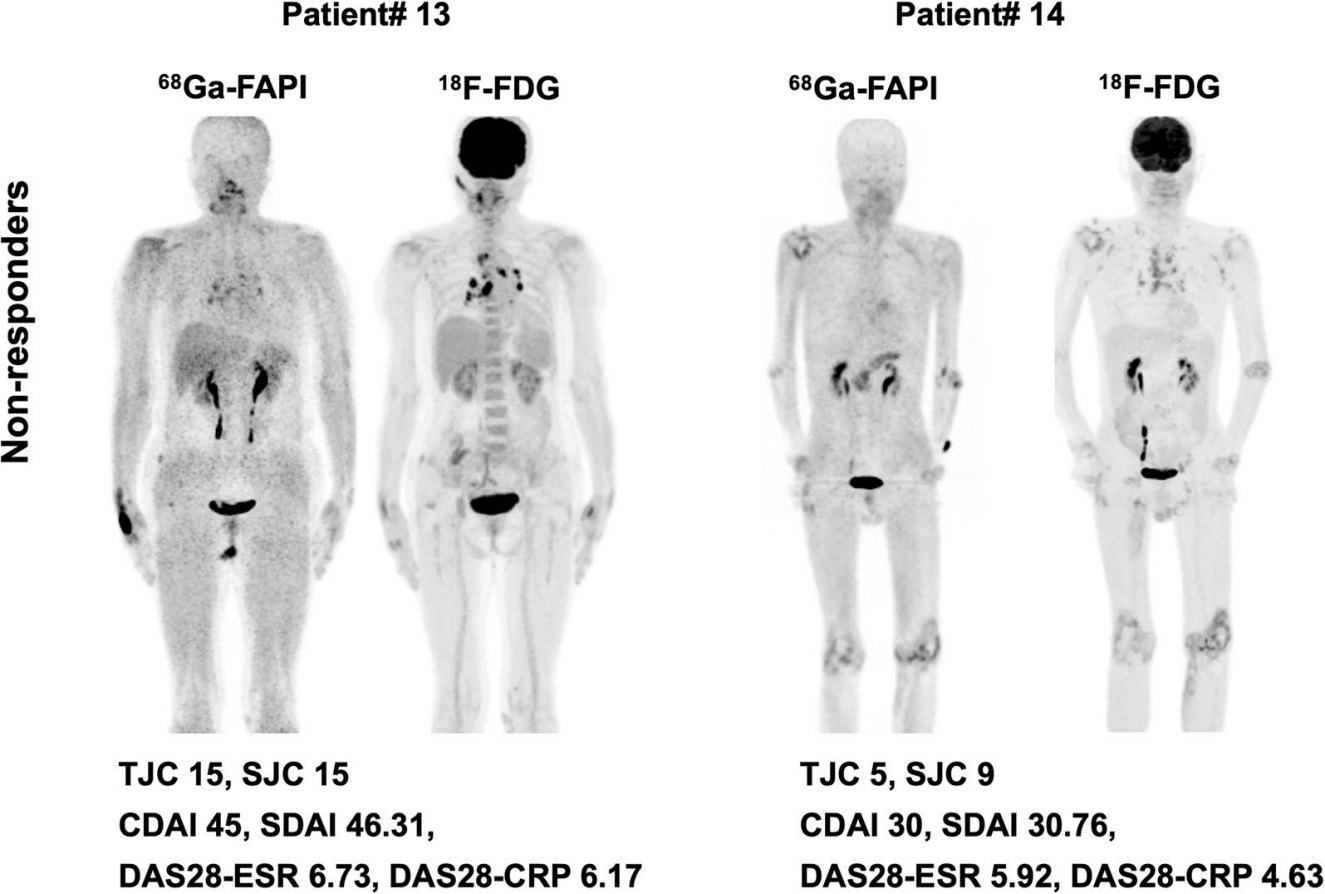
Representative [^68^ Ga]Ga-FAPI-04 and [^18^F]FDG PET/CT images of non-responders at 3-months’ follow-up based on CDAI and SDAI response criteria. Patient# 13 was a 64-year-old woman with 3 affected joints detected in PET/CT (PJC_FAPI_ = 3, TSU_FAPI_ = 27.7 SUV bw*ml, MSV_FAPI_ = 9.2 mL, TSU_FDG_ = 4.3 SUV bw*ml, MSV_FDG_ = 1.5 mL). She was treated with methotrexate and etanercept. The CDAI and SDAI decreased 4.4% and 1.6% respectively from baseline at 3-months’ follow up. In patient# 14 (56-year-old man), [^68^ Ga]Ga-FAPI-04 PET/CT showed 15 affected joints (PJC_FAPI_ = 15, TSU_FAPI_ = 44.0 SUV bw*ml, MSV_FAPI_ = 15.1 mL, TSU_FDG_ = 47.5 SUV bw*ml, MSV_FDG_ = 16.3 mL). The CDAI and SDAI decreased 40.0% and 30.6% from baseline after 3 months treating with prednisone, Tripterygium wilfordii and ibuprofen (change to methotrexate and adalimumab later). *ESR* Erythrocyte sedimentation rate, *CRP* C-reactive protein, *TJC* Tender joint count, *SJC* Swollen joint count, *CDAI* Clinical Disease Activity Index, *SDAI* Simplified Disease Activity Index, *DAS28* Disease Activity Score with 28-joint counts, *PJC* PET joint count, *TSU* Total synovitis uptake, *MSV* Metabolic synovitis volume

When comparing baseline PET/CT features between the responders and non-responders at 3-months’ follow-up, both TSU and MSV in [^68^ Ga]Ga-FAPI-04 and [^18^F]FDG PET/CT of the responders were significantly higher than those in non-responders according to CDAI and SDAI response criteria (Table 3, *P* < 0.05). The PJC detected in [^68^ Ga]Ga-FAPI-04 and [^18^F]FDG PET/CT were also significantly higher in responders than non-responders according to CDAI response criteria (Table 3, *P* = 0.016 and 0.045, respectively). However, the clinical characteristics of disease activity at baseline (including ESR, tender or swollen joint count, VAS of pain, PGA, EGA, HAQ-DI, SF-36, CDAI, SDAI, DAS28-ESR, DAS28-CRP) did not show significant difference between the responders and non-responders, except that the CRP level in responders was significantly higher than that in non-responders (Table 3, *P* = 0.035 and 0.033 in CDAI and SDAI response criteria, respectively). At 6-months’ follow-up, the PET/CT features or clinical characteristics of disease activity did not show significant difference between patients with a good response (major or moderate response) and those with poor response (minor response or no response) (*P* > 0.05). The representative PET/CT images of responders and non-responders at 3-months were shown in Figs. 2 and 3.

**Table 3:** The comparison of baseline clinical and PET/CT characteristics between responders and non-responders at 3-months

		CDAI response			SDAI response		
		Responders (*N* = 10)	Non-responders (*N* = 9)	*P*	Responders (*N* = 11)	Non-responders (*N* = 8)	*P*
Clinical assessment of disease activity	ESR, mm/h	66.1 ± 25.3	48.7 ± 31.8	0.204	61.4 ± 28.7	53.1 ± 30.9	0.558
	CRP, mg/dL	5.6 ± 5.2	1.8 ± 2.0	0.035*	5.2 ± 5.0	1.8 ± 2.2	0.033*
	Tender or swollen joint count	17.7 ± 7.8	12.0 ± 5.2	0.081	16.6 ± 8.2	12.8 ± 5.0	0.252
	VAS of pain	6.6 ± 1.2	6.0 ± 1.6	0.357	6.5 ± 1.1	6.0 ± 0.6	0.409
	PGA	5.9 ± 1.1	6.4 ± 1.5	0.378	6.0 ± 1.1	6.4 ± 1.6	0.551
	EGA	6.6 ± 1.3	7.1 ± 0.8	0.346	6.5 ± 1.2	7.3 ± 0.7	0.206
	HAQ-DI	1.9 ± 0.7	1.4 ± 0.8	0.088	1.9 ± 0.7	1.4 ± 0.8	0.210
	SF36	46.1 ± 9.5	46.8 ± 9.8	0.882	46.1 ± 9.5	46.8 ± 9.8	0.882
	CDAI	33.2 ± 10.5	31.4 ± 9.5	0.709	32.1 ± 10.6	32.8 ± 9.3	0.899
	SDAI	38.8 ± 14.0	33.2 ± 10.7	0.352	37.3 ± 14.2	34.6 ± 10.7	0.647
	DAS28-ESR	6.4 ± 0.9	5.9 ± 0.9	0.361	6.2 ± 1.0	6.1 ± 0.9	0.900
	DAS28-CRP	6.0 ± 1.3	5.1 ± 1.1	0.119	5.9 ± 1.3	5.2 ± 1.2	0.219
^68^ Ga-FAPI PET/CT	PJC_FAPI_	15.5 ± 8.6	6.8 ± 4.8	0.016*	14.4 ± 8.9	7.3 ± 4.9	0.059
	TSU_FAPI_	803.7 ± 615.6	214.9 ± 234.6	0.015*	753.6 ± 607.2	210.2 ± 250.4	0.029*
	MSV_FAPI_	196.0 ± 150.9	58.6 ± 63.2	0.021*	183.7 ± 148.9	58.4 ± 67.6	0.041*
	SUVmax	10.1 ± 5.4	8.0 ± 3.6	0.342	10.4 ± 5.2	7.3 ± 3.1	0.155
^18^F-FDG PET/CT	PJC_FDG_	14.2 ± 9.6	6.6 ± 4.7	0.045*	13.2 ± 9.7	7.0 ± 4.8	0.117
	TSU_FDG_	433.1 ± 374.8	91.2 ± 148.4	0.021*	323.0(86.0, 614.1)	26.7(4.2, 125.4)	0.032*
	MSV_FDG_	121.8 ± 91.7	29.3 ± 46.4	0.014*	112.0 ± 92.8	31.1 ± 49.3	0.039*
	SUVmax	6.9 ± 3.2	4.8 ± 2.0	0.141	5.6(4.7, 7.5)	4.6(3.4, 5.4)	0.127

^*^P value is with statistically significancy

*ESR* Erythrocyte sedimentation rate, *CRP* C-reactive protein, *VAS* Visual analog scale, *PGA* Patient global assessment, *EGA* Evaluator global assessment, *HAQ-DI* Health Assessment Questionnaire Disability Index, *SF-36* Short Form-36., *CDAI* Clinical Disease Activity Index, *SDAI* Simplified Disease Activity Index, *DAS28* Disease Activity Score with 28-joint counts, *PJC* PET joint count, *TSU* Total synovitis uptake, *MSV* Metabolic synovitis volume

**Fig. 4:**
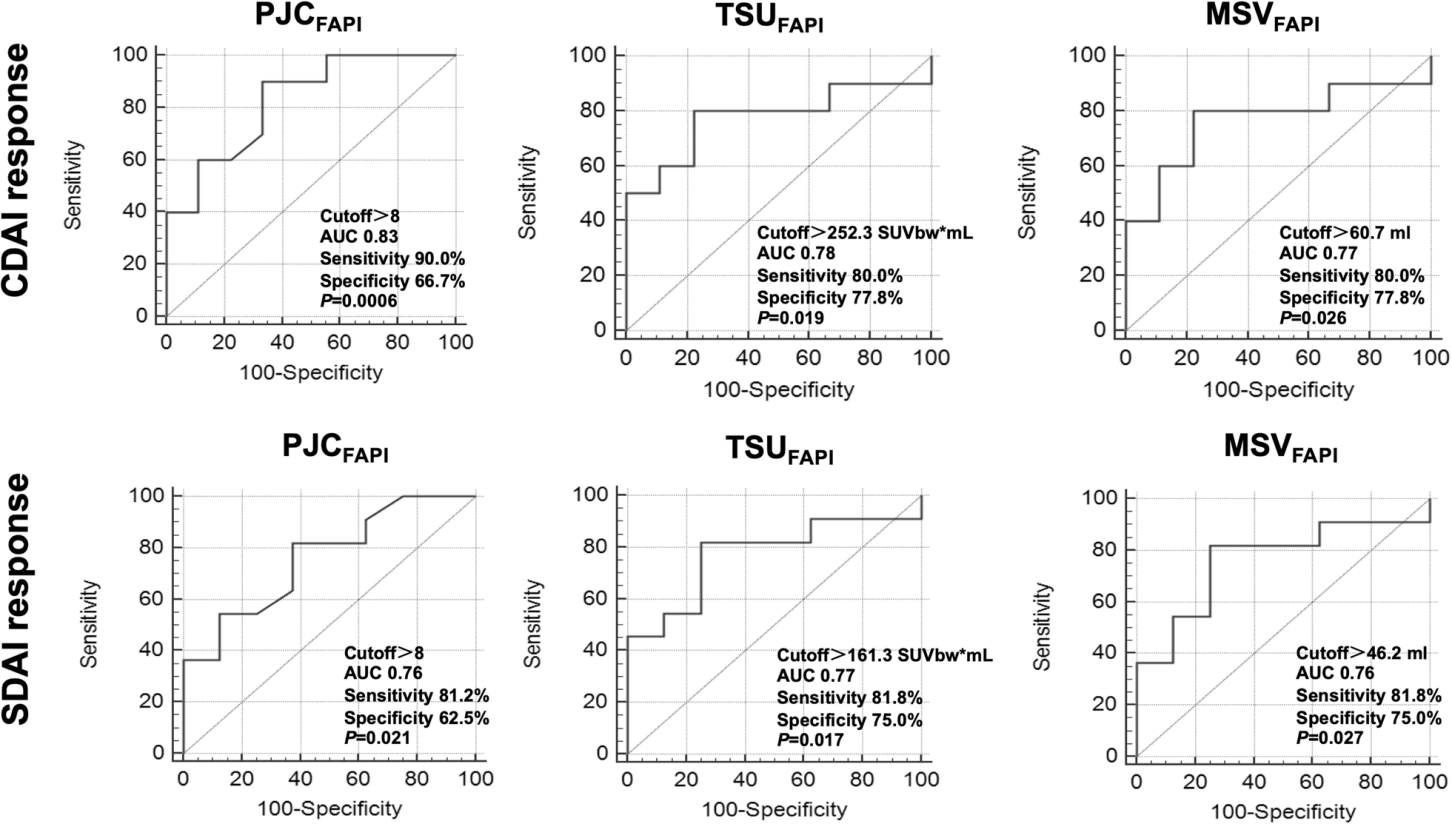
Receiver operating characteristic analysis curves of [^68^Ga]Ga-FAPI-04 PET/CT parameters for predicting 3-months’ CDAI and SDAI response

**Fig. 5:**
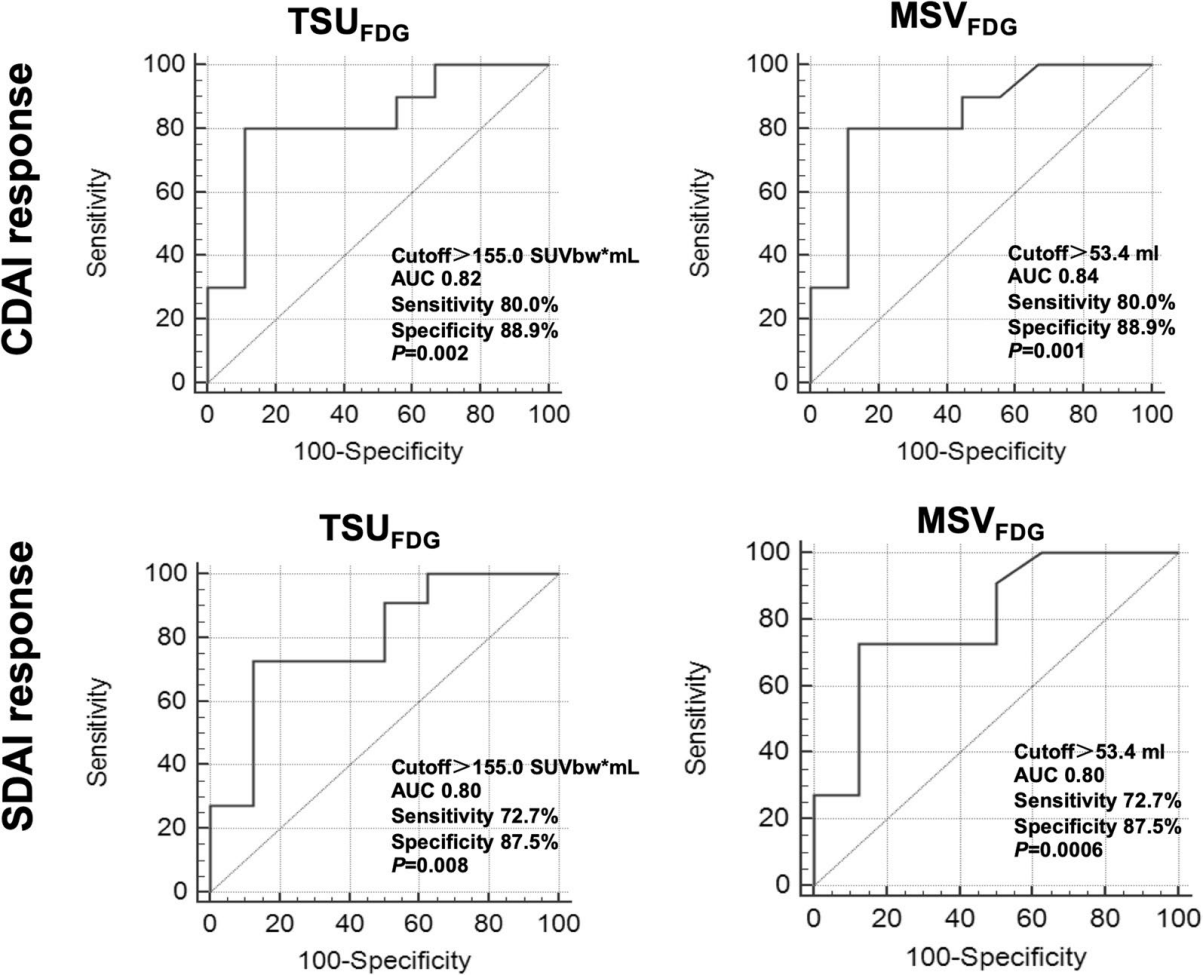
Receiver operating characteristic analysis curves of [^18^F]FDG PET/CT parameters for predicting 3-months’ CDAI and SDAI response

The ROC curves of [^68^ Ga]Ga-FAPI-04 and [^18^F]FDG PET/CT parameters were performed to differentiate responders and non-responders at 3-months’ follow-up. In [^68^ Ga]Ga-FAPI-04 PET/CT, PJC_FAPI_ > 8 (AUC 0.83 [0.59–0.96], sensitivity 90.0%, specificity 66.7%,* P* = 0.0006), TSU_FAPI_ > 252.3 SUVbw*mL (AUC 0.78 [95% CI 0.53–0.93], sensitivity 80.0%, specificity 77.8%*, P* = 0.019) and MSV_FAPI_ > 60.7 mL (AUC 0.77 [95% CI 0.52–0.93], sensitivity 80.0%, specificity 77.8%*, P* = 0.026) successfully discriminated CDAI responders and non-responders (Fig. 4). As to SDAI response, PJC_FAPI_ > 8 (AUC 0.76 [95% CI 0.51–0.92], sensitivity 81.8%, specificity 62.5%*, P* = 0.021), TSU_FAPI_ > 161.3 SUVbw*mL (AUC 0.77 [95% CI 0.53–0.93], sensitivity 81.8%, specificity 75.0%*, P* = 0.017) and MSV_FAPI_ > 46.2 mL (AUC 0.76 [95% CI 0.51–0.92], sensitivity 81.8%, specificity 75.0%*, P* = 0.027) also predicted the responders (Fig. 4). In [^18^F]FDG PET/CT, TSU_FDG_ > 155.0 SUV bw*mL (CDAI response, AUC 0.82 [95% CI 0.58–0.96], sensitivity 80.0%, specificity 88.9%*, P* = 0.002; SDAI response, AUC 0.80 [95% CI 0.55–0.94], sensitivity 72.7%, specificity 87.5%*, P* = 0.008) and MSV_FDG_ > 53.4 mL (CDAI response, AUC 0.84 [95% CI 0.60–0.97], sensitivity 80.0%, specificity 88.9%*, P* = 0.001; SDAI response, AUC 0.80 [95% CI 0.56–0.95], sensitivity 72.7%, specificity 87.5%*, P* = 0.006) could predict CDAI and SDAI responders (Fig. 5).

## Discussion

The rationale for treatment response prediction lies in the pathophysiological, clinical, and therapeutic heterogeneity of RA. In the current study, we found PJC, TSU and MSV of the synovitis detected in [^68^ Ga]Ga-FAPI-04 PET/CT before treatment had significant difference between responders and non-responders according to CDAI and SDAI response criteria at 3-months after initiation of tight control treatment. However, none of the clinical variables for assessing disease activity except CRP levels showed significant difference between responders and non-responders. Furthermore, we found those indices of [^68^ Ga]Ga-FAPI-04 PET/CT > cutoff values successfully predicted early responders in ROC curve analysis. [^68^ Ga]Ga-FAPI-04 PET/CT identified these differences in patients with different response status at baseline which preliminarily qualified it as a treatment response predictor.

Our results showed that the early responders had more involved joints shown in [^68^ Ga]Ga-FAPI-04 PET/CT and significantly higher TSU_FAPI_ and MSV_FAPI_ of the synovitis, although the responders and non-responders had similar clinical disease activity indexes and comparable counts of tender or swollen joints. The parameters derived from [^18^F]FDG PET/CT showed similar results to [^68^ Ga]Ga-FAPI-04 between the responders and non-responders, however [^18^F]FDG had a lower number of affected joints and uptake values compared with [^68^ Ga]Ga-FAPI-04. It is known that [^18^F]FDG uptake depends on the high glycolytic activity of inflammatory cells of synovitis. Meanwhile [^68^ Ga]Ga-FAPI-04 is a tracer targeting at FAP of the fibroblast-like synoviocytes [23], which play a key role in the development of RA through displaying an aggressive phenotype and pro-inflammatory effects by producing cytokines that activate B and T cells [24–26]. DMARDs, a class of anti-inflammatory and immunomodulatory agents, prevent and relieve RA aggression through suppressing the key inflammatory cells and synovial fibroblasts, proteins and inflammatory cytokines [27, 28]. Therefore we think higher uptake of [^68^ Ga]Ga-FAPI-04 and [^18^F]FDG before treatment suggested more activated inflammation of synovitis, thus indicated better reaction to systemic mediators against inflammation and better clinical response [29]. This speculation was also supported by our result that early responders had significantly higher level of CRP than non-responders, which was in accordance with previous studies [30, 31], and that TSU and MSV in [^68^ Ga]Ga-FAPI-04 and [^18^F]FDG PET/CT were positively correlated with CRP level.

When we further analyzed the PET positive joints and tender or swollen joint in physical examination, we found that the non-responders had significantly higher rate of joints that were tender or swollen but were PET negative (non-responders vs. responders, 44.0% vs. 12.4% in CDAI response, *P* < 0.0001; 43.7% vs. 13.7% in SDAI response, *P* < 0.0001). It may indicate that the non-responders have more secondary damage in the joints rather than active inflammation of synovitis, and their inflammatory activity was actually lower than it was revealed by physical examination. This overestimation of disease activity may lead to unsatisfactory response to anti-inflammation therapy. On the other side, we found that the responders had significantly higher rate of PET positive joints that were not tender or swollen in physical examination (responders vs. non-responders, 25.9% vs. 12.8% in CDAI response, *P* = 0.0081; 26.2% vs. 11.7% in SDAI response, *P* = 0.0037). It may suggest that some subclinical inflammation can only be detected by PET imaging rather than by physical examination. Early initiation of treatment is an essential strategy in treating RA, because joint damage, which may ultimately result in disability, begins early in the course of disease, and that the longer disease activity persists, the less likely the patient is to respond to therapy [32]. Thus physical examination, a less sensitive method to detect subclinical inflammation may not be an optimal method to evaluate disease activity.

Some studies showed that poor clinical response at 3-months’ time point makes achievement of low disease activity at 1 year unlikely [33], and good response at 6-months’ time point independently predicts significantly better 5-year clinical and radiographic outcome [34]. In our study, PET-derived parameters failed to predict 6-months’ response. As one patient was excluded from 6-months’ response analysis due to self-withdrawal of the drugs, and the treatment regimens were adjusted in some patients, further clinical trials are needed to find out whether [^68^ Ga]Ga-FAPI-04 PET/CT could predict late response, clinical remission after tight control treatment, clinical relapse, and radiographic progression.

As to the clinical markers for predicting treatment response, some studies found that rheumatoid factor and anti-cyclic peptide containing citrulline antibodies were closely associated with treatment response. The presence of rheumatoid factor or anti-cyclic peptide containing citrulline antibodies were reported to be associated with a good response to rituximab at 24 weeks, and were associated with better effectiveness of abatacept therapy, which may reflect a more specific autoimmune B cell and T cell reactivity allowing better efficacy of biologic DMARDs [35–37]. In our study, we did not find any association of rheumatoid factor and anti-cyclic peptide containing citrulline antibodies with treatment response (not shown in the results). It may because of the small cohort number as well as the fact that nearly all recruited patients in the current study having positive rheumatoid factor and anti-cyclic peptide containing citrulline antibodies.

To avoid some of the systemic immune suppressive effects of current therapies of RA, there has been renewed interest in the synovium-targeted therapy [38]. Recently, cyclin-dependent kinases inhibitors, that directly suppresses proliferation of fibroblast-like synoviocyte have demonstrated good efficacy and potency in preclinical models of arthritis, and the corresponding clinical trial in RA patients is ongoing [9–13]. There are also other candidate drugs that directly target fibroblast-like synoviocyte currently being studied [39, 40]. Another preclinical study showed the feasibility of depleting activated synovial fibroblasts through photodynamic therapy targeting fibroblast activation protein for locoregional therapy of RA [41]. Thus, there is increasingly need to establish a direct method to image fibroblast-like synoviocyte in RA patients. We think [^68^ Ga]Ga-FAPI-04 may have a greater role in patients treated with synoviocyte-targeted therapy.

Our study had several limitations. First, the cohort had a relatively small number of patients with a short-term follow-up. Second, the treatment included a spectrum of conventional, biological and targeted synthetic DMARDs. Prospective studies with specific therapeutic regimen should be conducted to further investigate the role of [^68^ Ga]Ga-FAPI-04 PET in the prediction and response evaluation. Third, because of the limited number of patients in our study, we did not further perform a multivariate logistic regression model to validate the potential predictors.

## Conclusion

[^68^ Ga]Ga-FAPI-04 demonstrated a greater number of affected joints and higher tracer uptake in RA compared with [^18^F]FDG. The PJC, TSU and MSV in [^68^ Ga]Ga-FAPI-04 PET/CT at baseline were significantly higher in early responders than those in non-responders based on CDAI and SDAI response criteria. The result suggested that [^68^ Ga]Ga-FAPI-04 PET/CT may predict the treatment response in RA patients.

## Data Availability

The datasets generated during and/or analysed during the current study are available from the corresponding author on reasonable request.

## Abbreviations

RA: Rheumatoid arthritis
DMARDs: Disease-modifying antirheumatic drugs
FAP: Fibroblast activation protein
FAPI: Fibroblast activation protein inhibitor
TJC: Tender joint count
SJC: Swollen joint count
ESR: Erythrocyte sedimentation rate
CRP: C-reactive protein
PGA: Patient global assessment
EGA: Evaluator global assessment
VAS: Visual analog scales
HAQ-DI: Health Assessment Questionnaire Disability Index
SF-36: Short Form-36
CDAI: Clinical disease activity index
SDAI: Simplified disease activity index
DAS28: Disease Activity Score using 28-joint counts
PJCPET: Joint count
MSV: Metabolic synovitis volume
TSU: Total synovitis uptake
[18F]FDG: 2- [18F]fluoro-2-deoxy-d-glucose
PET/CT: Positron emission tomography/computed tomography
SUVmax: Maximum standardized uptake value
